# Effects of revised diagnostic recommendations on malaria treatment practices across age groups in Kenya

**DOI:** 10.1111/j.1365-3156.2008.02072.x

**Published:** 2008-06

**Authors:** D Zurovac, J Njogu, W Akhwale, D H Hamer, B A Larson, R W Snow

**Affiliations:** 1 Malaria Public Health and Epidemiology Group, KEMRI/Wellcome Trust Research ProgrammeNairobi, Kenya; 2 Centre for Tropical Medicine, University of OxfordOxford, UK; 3 Ministry of Health, Division of Malaria ControlNairobi, Kenya; 4 Department of International Health, Boston University School of Public HealthBoston, MA, USA; 5 Section of Infectious Diseases, Boston University School of MedicineBoston, MA, USA

**Keywords:** malaria, diagnostics, artemether-lumefantrine, guidelines, Kenya

## Abstract

**Objective:**

The recent change of treatment policy for uncomplicated malaria from sulfadoxine-pyrime-thamine to artemether-lumefantrine (AL) in Kenya was accompanied by revised malaria diagnosis recommendations promoting presumptive antimalarial treatment in young children and parasitological diagnosis in patients 5 years and older. We evaluated the impact of these age-specific recommendations on routine malaria treatment practices 4–6 months after AL treatment was implemented.

**Methods:**

Cross-sectional, cluster sample survey using quality-of-care assessment methods in all government facilities in four Kenyan districts. Analysis was restricted to the 64 facilities with malaria diagnostics and AL available on the survey day. Main outcome measures were antimalarial treatment practices for febrile patients stratified by age, use of malaria diagnostic tests, and test result.

**Results:**

Treatment practices for 706 febrile patients (401 young children and 305 patients ≥5 years) were evaluated. 43.0% of patients ≥5 years and 25.9% of children underwent parasitological malaria testing (87% by microscopy). AL was prescribed for 79.7% of patients ≥5 years with positive test results, for 9.7% with negative results and for 10.9% without a test. 84.6% of children with positive tests, 19.2% with negative tests, and 21.6% without tests were treated with AL. At least one antimalarial drug was prescribed for 75.0% of children and for 61.3% of patients ≥5 years with a negative test result.

**Conclusions:**

Despite different recommendations for patients below and above 5 years of age, malaria diagnosis and treatment practices were similar in the two age groups. Parasitological diagnosis was under-used in older children and adults, and young children were still tested. Use of AL was low overall and alternative antimalarials were commonly prescribed; but AL prescribing largely followed the results of malaria tests. Malaria diagnosis recommendations differing between age groups appear complex to implement; further strengthening of diagnosis and treatment practices under AL policy is required.

## Introduction

Antimalarial treatment with highly efficacious but expensive artemisinin-based combination therapy (ACT) is a recent key strategy to reduce the public health impact of failing monotherapies in sub-Saharan Africa (White 2006; WHO 2006). In 2004, Kenya changed its first-line treatment policy for uncomplicated malaria from sulfadoxine-pyrimethamine (SP) to a specific ACT, artemether-lumefantrine (AL). Quinine became the treatment of choice for children below 5 kg, pregnant women, and as the second-line; SP was reserved only for intermittent preventive treatment in pregnancy; and amodiaquine (previous second-line treatment) was no longer recommended (MoH 2006a).

To rationalize the use of expensive ACTs, the WHO promotes parasitological diagnosis, except for young children in high malaria risk areas where, pending further evidence, the clinical consequences associated with not treating potentially false negative test results may outweigh potential cost-saving benefits (World Health Organisation (WHO) 2006). To support implementation of the new AL policy, the Kenyan Ministry of Health (MoH) revised recommendations for malaria diagnosis at facilities where malaria microscopy or rapid diagnostic tests (RDT) are available. Considering age-specific risks to clinical consequences of malaria, age-specific probability of malaria exposure, and the cost of AL treatment across different age groups, the new diagnosis recommendations were reflected in revised guidelines (Ministry of Health (MoH) 2006a). In summary, the guidelines recommend that all febrile children below 5 years of age in high malaria risk areas should be presumptively treated with AL. All parts of Kenya are classified as high malaria risk areas, except the highlands of Central and Nairobi provinces. All febrile patients without another obvious cause of fever aged ≥5 years should have a malaria test performed, and health workers should treat for malaria only patients who test positive.

In 2006 the new AL policy was implemented countrywide and evaluated approximately 4–6 months after AL was delivered to health facilities. Descriptions of the implementation process and overall paediatric malaria case-management results were published earlier (Amin *et al.* 2007; Zurovac *et al.* 2008). We report here how age-specific malaria diagnosis recommendations were translated into routine malaria treatment practices at facilities with diagnostic capacity.

## Methods

Between October and December 2006, we evaluated outpatient malaria case-management at government health facilities in four Kenyan districts (Kwale, Bondo, Kisii/Gucha and Makueni). The detailed explanation of survey methods is presented elsewhere (Zurovac *et al.* 2008). Briefly, the survey was a cross-sectional, cluster sample survey undertaken at all 193 government facilities in the four districts. Data at each facility were collected over 1 day using quality-of-care assessment methods including health facility assessments, health worker interviews, and exit interviews with caretakers and patients. During the exit interviews the study nurses collected information about patient's age, weight, temperature, history of fever, pregnancy status, main complaints, prior use of antimalarial drugs and if the visit was an initial or follow-up consultation. Information was also collected from patient-held records about routine diagnostic procedures requested and results reported, medications prescribed, and if the patient was treated as an outpatient or referred for hospitalization. The present analysis focused on routine antimalarial treatment practices for patients weighing ≥5 kg presenting for an initial consultation and on outpatient visit with a history of fever or axillary temperature ≥37.5 °C. Pregnant women and patients ≥5 years presenting with another obvious cause of fever were excluded from analysis. Since malaria diagnosis recommendations differ between age groups, we stratified this analysis for patients below and above 5 years of age. Treatment practices were further stratified by use and results of malaria tests. Given the small number of patients at facilities with malaria RDTs, which precludes a meaningful analysis stratified by type of diagnostics, the combined results from all facilities are presented. To ensure comparable evaluation of treatment practices, the analysis was restricted to facilities where AL was available on the survey day. The precision of proportions [95% confidence interval (CI)] was determined adjusting for the cluster sampling.

## Results

Of 193 health facilities assessed, 70 (36.3%) had parasitological capacity for malaria diagnosis, more commonly providing malaria microscopy (55/70, 78.6%) than RDTs (19/70, 27.1%). Only four facilities had both diagnostic capacities. We analysed treatment practices for 706 febrile patients (401 < 5 years and 305 ≥ 5 years of age) presenting to the 64 facilities with diagnostic support where AL was in stock on the survey day. Of these patients, the majority in each respective age group was evaluated at facilities with microscopy (88.0% and 84.9% respectively). Among patients ≥5 years, only 43.0% (95% CI: 34.4–51.5) had a diagnostic test performed. At the same facilities, 25.9% of children <5 years of age (95% CI: 16.8–25.1) were tested. Positive malaria tests were routinely reported for 50.0% of children (95% CI: 39.3–60.7) and 52.7% of patients ≥5 years (95% CI: 42.8–62.5).

The pattern of antimalarial treatments was similar among febrile patients below and ≥5 years (Table 1). AL was prescribed for 84.6% (95% CI: 71.5–97.7) of children with positive test results, for 19.2% (95% CI: 5.8–32.7) with negative results, and for 21.6% (95% CI: 8.3–34.8) of children who were not tested. Among patients ≥5 years, 79.7% (95% CI: 64.1–95.3) of patients with positive tests were treated with AL, while 9.7% (95% CI: 0.2–19.2) with negative tests and 10.9% (95% CI: 4.8–17.0) without a test were also treated with AL. Notably, in both age groups, antimalarial treatment was prescribed for the majority of patients with negative test results [75.0% (95% CI: 60.2–89.8) of children and 61.3% (95% CI: 47.0–75.6) of patients ≥5 years] and those without tests performed [64.3% (95% CI: 54.9–73.7) of children and 50.0% (95% CI: 39.4–60.6) of patients ≥5 years]. Across all patient categories, except those with positive test results, amodiaquine and SP were the most commonly prescribed antimalarial treatments and over 80% of patients were treated with antibiotics (Table 1).

**Table 1:** Antimalarial treatment practices for febrile patients stratified by age and result of malaria test

	Children below 5 years of age				Patients 5 years and older			
		
	Positive test *n* = 52(%)	Negative test *n* = 52(%)	Test not done *n* = 297(%)	Total *n* = 401(%)	Positive test *n* = 69(%)	Negative test *n* = 62(%)	Test not done *n* = 174(%)	Total *n* = 305(%)
AL	44 (84.6)	10 (19.2)	64 (21.6)	118 (29.4)	55 (79.7)	6 (9.7)	19 (10.9)	80 (26.2)
AQ	5 (9.6)	18 (34.6)	97 (32.7)	120 (29.9)	3 (4.4)	17 (27.4)	37 (21.3)	57 (18.7)
SP	0	5 (9.6)	4 (1.4)	9 (2.2)	0	8 (12.9)	18 (10.3)	26 (8.5)
SP+AQ	1 (1.9)	5 (9.6)	14 (4.7)	20 (5.0)	1 (1.5)	4 (6.5)	13 (7.5)	18 (5.9)
QN	1 (1.9)	0	5 (1.7)	6 (1.5)	4 (5.8)	2 (3.2)	0	6 (2.0)
Other AM	0	1 (1.9)	7 (2.4)	8 (2.0)*	5 (7.3)	1 (1.6)	0	6 (2.0)†
No AM prescribed	1 (1.9)	13 (25)	106 (35.7)	120 (29.9)	1 (1.5)	24 (38.7)	87 (50.0)	112 (36.7)
Any AM prescribed	51 (98.1)	39 (75.0)	191 (64.3)	281 (70.1)	68 (98.6)	38 (61.3)	87 (50.0)	193 (63.3)
Antibiotic prescribed	32 (61.5)	44 (84.6)	250 (84.2)	326 (81.3)	41 (59.4)	54 (87.1)	137 (78.7)	232 (76.1)

AL, artemether-lumefantrine; AQ, amodiaquine; SP, sulfadoxine-pyrimethamine; QN, quinine; AM, antimalarial.

* Other antimalarial treatments include QN+AL (3), AQ+QN (2), dehydroartemisinin (2) and artemether (1).

† Other antimalarial treatments include QN+AL (1), AQ+QN (4) and dehydroartemisinin (1).

## Discussion

Kenya's policy to promote presumptive malaria diagnosis in young children and parasitological testing in patients ≥5 years should have resulted in substantially different patterns of malaria diagnostic and treatment practices between these age groups. However, the only difference found was somewhat higher use of malaria testing in patients ≥5 years (43%) than in young children (27%). In both age groups, any antimalarial drug was prescribed to the majority of patients with a positive test (98–99%), negative test (61–75%) and those without test performed (50–64%). AL, however, was rarely prescribed for all patients’ categories (10–22%), except for those with a positive test result (80–85%).

The observed pattern is likely to reflect modalities of AL implementation process in Kenya. Prior to the introduction of ACTs, several studies in Kenya (Zurovac *et al.* 2006a) and elsewhere (Barat *et al.* 1999; Holtz & Kachur 2000; Reyburn *et al.* 2007) reported high rates of negative test results treated with an antimalarial drug. As a response to the concern that the continuation of this practice under AL policy would waste new expensive drugs, particularly in older age groups (Zurovac *et al.* 2006b), the new guidelines unambiguously discouraged this practice for patients ≥5 years (Ministry of Health (MoH) 2006a). Simultaneously, prompt and effective presumptive treatment of young febrile children was promoted in line with international recommendations (Gove 1997; World Health Organisation (WHO) 2006). However, the translation of new guidelines into clinical practice faced several challenges. Firstly, although the guidelines recommended the use of malaria microscopy or RDTs for patients ≥5 years, there was no decision taken to procure and supply RDTs on larger scale. Secondly, the training of health workers greatly emphasized the high cost of AL demanding its rational use for test-positive adults using microscopy or RDTs, with less focus on providing prompt AL treatment for febrile children. Indeed, all health workers had been trained how to perform RDTs, their potential in peripheral facilities had been emphasized and even limited quantities of RDTs were delivered for training purposes (MoH 2006b). Thirdly, training messages commonly included incorrect information about expecting RDT supplies, compulsory testing before prescribing AL for all age groups, and adequate efficacy of non-recommended antimalarials (Wasunna *et al.* 2008). Finally, the national guidelines were somewhat ambiguous as they stated ‘*parasitological diagnosis is not prerequisite for the treatment*’ in febrile children. Therefore, it was not surprising that we found similar age-specific practices characterized by suboptimal use of malaria diagnostics and prescription of AL predominantly for the small subset of test positive patients.

Antimalarial treatment practices for test negative older children and adults deserve special attention. Although the use of malaria diagnostics was low, our previous concerns that health workers would massively overprescribe AL for these patients appear to be unfounded (Zurovac *et al.* 2006a,b; Reyburn *et al.* 2006). The change of policy to AL has not eliminated unnecessary use of other inexpensive antimalarial drugs, but the new policy, however, had an impact on rational use of AL in this patient group. Although this is an encouraging finding, it should be cautiously interpreted since the pattern might change with longer establishment of AL policy, removal of amodiaquine from the facilities, and more widespread availability of RDTs as demonstrated in Zambia (Hamer *et al.* 2007).

## Conclusion

The translation of revised diagnostic recommendations into effective malaria treatment practices under the AL policy in Kenya faces several challenges. Parasitological diagnosis is underused in older children and adults, young children are still tested and the overall use of AL is low in both age groups with marked tendency for alternative antimalarial prescriptions. Prescribing of AL, however, largely followed malaria test results. Different age-specific diagnosis recommendations appear complex to implement. The most suitable solution for policy implementers would be to have the same recommendations for all age groups. RDTs are a potential solution for young children, as suggested recently in urban setting in Uganda (Njama-Meya *et al.* 2007); however more evidence across different settings is required to document if not treating test-negative children in high risk areas can produce significant cost-savings without resulting in harmful clinical consequences. Meanwhile, the priority for the Kenyan MoH should be strengthening of AL implementation activities based on presumptive treatment for young children and parasitological diagnosis, including RDTs, for patients 5 years and older.
